# Two Deep Image Reconstructions for a 320-Row CT: Review of Clinical Applications

**DOI:** 10.2174/0115734056429078260131131101

**Published:** 2026-02-17

**Authors:** Wanhui Zhou, Sihua Zhong, Jing Li, Guozhi Zhang

**Affiliations:** 1 Collaborative Innovation, United Imaging Healthcare, Shanghai 201807, China

**Keywords:** Computed tomography, Image quality, Deep learning reconstruction, Deep iterative reconstruction, Iterative reconstruction, Artificial intelligence, Radiation dose reduction, Clinical applications

## Abstract

With the increasing popularity and clinical adoption of deep learning in CT image reconstruction, two distinct approaches have emerged under the concept of 'deep' reconstruction: Deep Learning Reconstruction (DLR) and Deep Iterative Reconstruction (DIR). Despite falling under the umbrella of 'deep' reconstruction, DLR and DIR differ in technical principle, clinical applicability, and reconstruction performance. This review aims to provide a clinically oriented overview of these two methods, emphasizing their coexistence and differentiated roles on a 320-row CT scanner platform, offering radiologists insights for clinical practice as well as inspirations for future research. On this platform, DLR and DIR represent complementary strategies in clinical practice, where DLR is implemented as a cardiac-specific algorithm and DIR for other bodyparts. By summarizing representative clinical applications, we highlight the advantages of DLR in cardiac CT and strengths of DIR across chest, abdominal, vascular, and perfusion CT imaging. Quantitative evidence from recent studies demonstrates consistent improvements of both DIR and cardiac-specific DLR over routine hybrid iterative reconstruction. Their complementary characteristics also suggest potential benefits when applied in multi-region CT imaging. In addition, the clinically valuable image features of DIR that merit further investigation, as well as other technical considerations relevant to 'deep' reconstructions are discussed.

## 1 INTRODUCTION

In the past few decades, as advances in Computed Tomography (CT) hardware continue to expand the boundaries of physical limits, parallel developments in image recons-truction algorithms have enabled further improvement in the diagnostic performance of CT. Hybrid Iterative Reconstruction (HIR), which reduces noise and artifacts by performing corrections in both the projection and image domains, has replaced Filtered Back Projection (FBP) to serve as the current clinical standard [1].

As clinical demands grow for lower radiation doses and improved spatial resolution, the performance of the current routine reconstruction algorithm, HIR, is also reaching its limits [2], particularly in challenging clinical tasks like ultra-low-dose screening [3] and low-contrast lesion detection [4]. Model-Based Iterative Reconstruction (MBIR) provides superior capabilities to HIR on noise reduction, artifact suppression, and spatial resolution improvement. However, the unnatural image appearance related to its high-strength level and the long reconstruction time resulted from its sophisticated algorithm structure, limited its clinical application [1, 5]. These limitations have prompted the development of more advanced reconstruction techniques.

With the emergence and rapid development of deep learning techniques, the field of CT reconstruction has entered a new era, marked by the integration of Convolutional Neural Network (CNN) into the image formation process [6]. Depending on how the CNN is integrated into the reconstruction workflow, two conceptually distinct approaches have emerged and are now available on the same CT platform: Deep Learning Reconstruction (DLR) and Deep Iterative Reconstruction (DIR). DLR, also called deep learning-based reconstruction or deep learning image reconstruction, has been commercially implemented by several CT vendors [6]. As for DIR, which integrates CNN into the MBIR workflow, its technical complexity has posed challenges for widespread commercial implementation. The Artificial Intelligence Iterative Reconstruction (AIIR, United Imaging Healthcare) stands as a pioneering clinical implementation of DIR [7].

Both DLR and DIR have demonstrated significant advantages in clinical CT imaging as compared to HIR, including dose reduction [8, 9], spatial resolution enhancement [10, 11], and artifact suppression [9, 12]. Despite falling under the broad concept of 'deep' reconstruction, DLR and DIR differ substantially in many aspects. Along with their increasing prevalence, they are currently available on the latest generation 320-row detector CT scanner (uCT ATALAS, United Imaging Healthcare) and utilized for different clinical scenarios: DLR for cardiac applications and AIIR for others.

This review aims to present a comprehensive and clinically oriented overview of the two 'deep' reconstructions. Given that the two methods coexisting on the 320-row CT platform are designed for distinct clinical scenarios, we emphasize their complementary roles and respective strengths over HIR by presenting a series of clinical cases, rather than directly comparing them. In addition, we also present discussions on technical perspectives and future directions.

## 2 TECHNICAL BACKGROUND

### 2.1 Emergence of 'Deep' Reconstructions

As representative examples of vendor-specific DLR, TrueFidelity (GE Healthcare) [13] and Precise Image (Philips Healthcare) [14] adopt a projection-domain strategy, operating directly on the projection data to generate CT images. In contrast, some vendor-specific DLR, like Advanced Intelligent Clear-IQ Engine (AiCE) [15] and Precise IQ Engine (PIQE) (Canon Medical Systems) [16], as well as vendor-agnostic PixelShine and ClariCT.AI, are image-domain implementations, functioning as a post-processing denoiser applied to FBP or iterative images. Their clinical performance has been evaluated across numerous published studies [11, 17-22]. Existing literature reviews have also summarized their developing evolvement, compared their technical principles, and reviewed their clinical applications according to varying dose levels, imaging protocols, and clinical tasks [2, 5, 6, 20]. However, DIR is fundamentally different from DLR in its design and implementation. Unlike DLR, DIR only operates on projection data. Despite growing investigations on its clinical performance [10, 23-28], DIR has not been comprehensively reviewed in prior literature, and the relationship with DLR remains indistinct.

### 2.2 Two 'Deep' Reconstructions Coexisting on a 320-row CT Platform

While previous studies have assessed each approach independently, understanding their technical distinctions is essential for interpreting their clinical performance. This section, therefore, illustrates the technical background of DLR and DIR and describes the differences. At the time of writing, a 320-row CT scanner platform (uCT ATALAS, United Imaging Healthcare) provides both DLR and DIR, whose basic concept diagram is shown in Fig. (**1**) and detailed parameters are summarized in Table **1**. All images shown in this article were acquired from this CT platform. The reconstruction system includes the HIR (Karl 3D, United Imaging Healthcare) as the baseline, alongside the advanced DLR and DIR modules available. The DLR module utilizes the reconstruction algorithm, CardioBoost (formerly referred to as DELTA [9]). The DIR module utilizes the AIIR algorithm. CardioBoost is specifically optimized for cardiac application during the training process, while AIIR is trained for all other body regions. These 'deep' reconstructions are executed on a standalone workstation with multiple GPUs, enabling highly efficient reconstruction for both DLR and DIR through parallel processing.

#### 2.2.1 DLR: CardioBoost

CardioBoost is a projection-domain DLR where the deep learning model is trained to output CT images directly from the projection data and to replace traditional reconstructions. CardioBoost achieves high-quality image reconstruction directly from low-quality sonogram data input through a U-shaped network. The network employs several residual modules to efficiently learn the training data and mitigate undesirable noise and artifacts. Based on the standard-dose images as the ground truth, the corresponding low-dose projection data is built with a low-dose simulation algorithm [29] by adding noise into the projection domain of standard-dose data. The low-dose data is simulated based on the standard-dose data to avoid second scanning and avoid data mismatch due to motion between the two scans. CardioBoost is tailored for cardiac applications [9] with the training dataset covering typical cardiac clinical cases, including coronary artery stenosis, coronary anomalies, implanted cardiac devices, *etc*. In order to optimize cardiac CT imaging, a deep learning-based motion correction module pre-trained for cardiac motion compensation has also been introduced in combination with CardioBoost for correcting motion artifacts.

#### 2.2.2 DIR: AIIR

DIR refers to an innovative type of 'deep' reconstruction that integrates the CNN into the iterative reconstruction process. AIIR has a unique architecture combining the advantages of MBIR and deep learning [20, 24, 30]. As shown in Fig. (**1b**), the architecture of AIIR is based on the framework of MBIR, where a data-driven deep learning de-noising engine is integrated and replaces the conventional regularization function. Altogether with the MBIR framework and the deep learning de-noising engine, the image quality is improved during the iterations where the detailed information is preserved, the noise and artifact is suppressed, and the natural image texture is maintained. The training dataset of the de-noising engine contains millions of high-quality and low-dose image pairs, which are both reconstructed using the MBIR algorithm. The dataset covers various clinical scenarios, including multiple body parts, scan protocols, and radiation dose levels, to ensure the generalizability and robustness of the network. With training datasets covering multiple body parts except for cardiac datasets, AIIR is not intended for a certain body part and thus is applicable in a variety of clinical scenarios.

## 3 CLINICAL APPLICATIONS

In this section, clinical potentials of the two 'deep' reconstructions are presented by outlining the unmet clinical needs and illustrating representative clinical cases across diverse clinical scenarios. Quantitative metrics measured on these images are summarized in Table **2**. In addition, a list of related studies can be found in Table **3**.

### 3.1 Cardiac CT: Improving Spatial Resolution for Both Coronary Artery and Cardiac Structures

The fundamental requirement for cardiac CT is to acquire motion-free images of the entire heart during peak contrast enhancement, allowing optimal visualization of coronary arteries and cardiac structures. Beyond motion correction, achieving high spatial resolution without increasing image noise is essential for accurate assessment of vessel stenosis and other structural abnormalities. However, spatial resolution, image noise, and radiation dose are inherently linked, and improving one often compromises the others [11]. Thus, the implementation of image reconstruction techniques that enable improving spatial resolution could be an attractive solution.

DLR and DIR differ significantly in technical principles, which impact their applicability on cardiac CT. DLR, particularly the CardioBoost, which is compatible with the deep learning-based motion correction network, has the potential to effectively suppress motion artifacts for rapidly moving structures. DLR also enhances spatial resolution and reduces noise simultaneously, making it particularly suitable for cardiac CT. Additionally, its fast reconstruction and workflow efficiency is advantageous in urgent scenarios, such as patients with acute chest pain, where immediate image availability is critical. In contrast, DIR relies on model-based iterative computations and is not compatible with the motion correction network, resulting in limited capability for cardiac motion suppression and workflow efficiency.

**Fig. (2):**
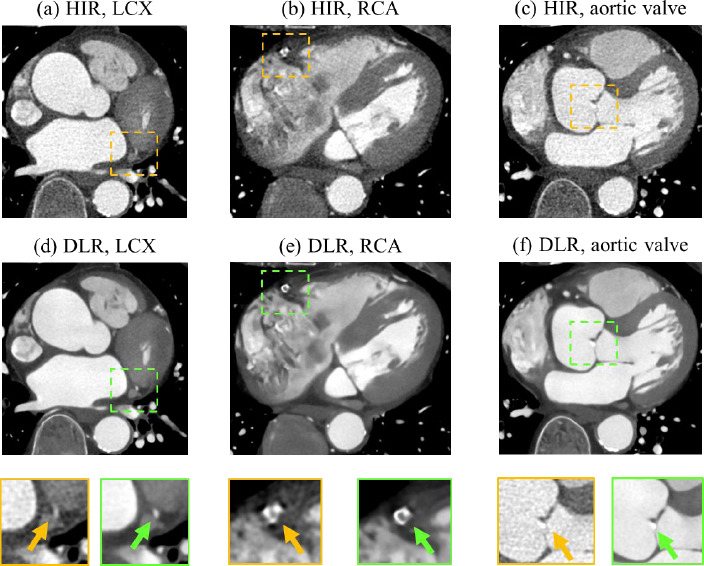
Coronary CTA images obtained in a 75-year-old woman with arrhythmia (average heart rate, 79 bpm). Images were acquired at a CTDI_vol_ of 17.8 mGy and reconstructed with HIR (**a~c**) and DLR (**d~f**). On DLR images, the distal segment of the Left Circumflex (LCX) is more conspicuous than on HIR images owing to the considerable reduction of image noise (a and d). The aortic valve structures, especially the leaflet, are more clearly visualized on DLR images, where the margin is blurred by the noise on HIR images. The calcified plaques in the Right Coronary Artery (RCA, b and e) and aortic valve (c and f) are more sharply delineated by DLR, contributing to the reduced partial volume and blooming artifacts.

With the cardiac-specific DLR, image noise can be remarkably reduced, especially at low-dose levels, while also minimizing motion artifacts and vessel blurring, as reported previously [9, 31]. DLR is also reported to enable reliable coronary artery calcium scoring with up to 82.7% lower radiation dose *via* simultaneously reduced tube voltage and current, while exhibiting a higher Contrast-To-Noise Ratio (CNR) than routine-dose HIR images. On routine-dose level, DLR could further improve the image quality with higher spatial resolution and lower noise magnitude while preserving noise texture, which facilitates more accurate assessment of both coronary and extra-coronary cardiac abnormalities (Fig. **2**). DLR improves the visualization of small coronary arteries, such as distal branches, by reducing edge blurring and enhancing contrast with surrounding tissues. It also minimizes blooming artifacts and partial volume effects around calcified plaques, enabling more accurate stenosis evaluation. Furthermore, DLR enhances visualization of cardiac valves and chamber morphology, supporting more reliable measurements for surgical planning, such as valve replacement.

The cardiac-specific DLR provides a balanced solution for achieving high spatial resolution, robust motion suppression, and dose optimization. These characteristics make it a powerful tool for cardiac CT imaging in clinical practice.

### 3.2 CT Angiography: From Suppressing Noise to Improving Details

In clinical practice, the visualization of small vessels in CT Angiography (CTA), particularly distal segments, can be challenging due to their small diameter and insufficient contrast medium. A major contributing factor is the limited spatial and contrast resolution of CT images with the routinely used HIR algorithm, which is dependent on the contrast and noise levels of the surrounding structures [32]. The emergence of advanced 'deep' reconstructions, especially DIR, offers a powerful solution by improving spatial resolution while reducing image noise. These capabilities enhance the visualization of fine vascular structures, even in low-dose settings.

It has been reported that DIR outperforms HIR in depicting pulmonary vasculature and emboli at routine dose, with reducing image noise by 35%~45% and improving Signal-To-Noise Ratio (SNR) and CNR by 70%~90% [23]. In low-dose settings, DIR also excels in visualizing fine structures of aortic lesions, yielding significantly reduced image noise, and approximately three-fold higher SNR and CNR, as compared to HIR [24]. As demonstrated in Fig. (**3**), DIR enhances the visualization of aortic dissection with improved spatial resolution, enabling more accurate diagnosis.

While DIR demonstrates superior performance in visualizing vascular details, the clinical relevance of these improvements may not matter in all clinical scenarios. The trade-off between high-computational-demand DIR processing and incremental gains in spatial resolution is the key consideration, according to the specific clinical tasks.

### 3.3 Chest CT: From Routine to Low and then to Ultralow Dose

Chest CT is one of the radiological fields that benefits the most from the advanced 'deep' reconstructions. Low-dose CT, enabled by 'deep' reconstructions, is becoming more and more popular as the routine screening method for lung cancer, achieving an effective dose as low as 1.22±0.34 mSv [33]. These algorithms enable substantial noise reduction while pre-serving anatomical details and detection sensitivity, making it well-suited for detecting bronchiectasis, honeycombing, and pulmonary nodules [34, 35]. As the diagnostic performance of low-dose CT is well established, there is growing interest in ultra-low-dose chest CT, aiming to reduce radiation exposure comparable to chest X-ray radiography [33]. This is particularly important for screening imaging, pediatric imaging, and follow-ups of chronic conditions where repeated scans are necessary.

On the 320-row CT platform, the availability of DIR has made ultra-low-dose chest CT feasible. Studies have shown that DIR maintains the ultra-low-dose chest CT image sufficient for clinical diagnosis. As reported by Zhang *et al.*, DIR reduced the image noise by ~64% as compared with HIR at the same low-dose level, which was reduced to approximately 0.1mSv, allowing reliable diagnosis for pediatric pneumonia and airway stenosis [36]. Besides, Hao *et al*. reported that DIR enabled accurate bone mineral density measurement on low-dose chest CT, which was called the “one-stop” screening protocol of lung cancer and osteoporosis, with reduced image noise and improved spatial resolution as compared with HIR [25].

An example of ultra-low-dose chest CT, of which the dose level at 0.13 mSv is comparable to the conventional chest X-ray radiography, is shown in Fig. (**4**). DIR obviously enhances image quality by reducing noise, suppressing artifacts, and preserving texture as compared with HIR. As such, DIR shows the ability to provide high-resolution lung images with increased visibility and sharper edges of subtle anatomic structures such as pulmonary nodules, vessels, and airways.

### 3.4 Abdominopelvic CT: Optimizing Tumor Characterization

A challenging scenario of abdominopelvic CT imaging is preoperative tumor imaging, where accurate tumor characterization often takes precedence over concerns about radiation dose. When assessing the primary tumor, local invasion, or distal metastasis, the diagnostic accuracy heavily relies on the image quality, particularly the contrast resolution between soft tissues.

While HIR is currently used as a clinical standard, it is often insufficient for differentiating subtle soft-tissue contrasts in complex oncologic scenarios. In these situations, DIR is particularly advantageous due to its physics-based modeling and deep learning-based denoising. It is reported that DIR excels in preserving fine anatomical details while suppressing noise and artifacts, thus improving contrast resolution and spatial resolution [8, 10]. As reported by Li *et al*., DIR provides superior image quality over HIR, characterized by significantly higher SNR and CNR, and more conspicuous imaging features, thus improving diagnostic accuracy in identifying peritoneal invasion and hepatic metastases in patients with colorectal cancer [10]. Besides, it is also reported that DIR outperforms HIR in SNR, CNR, and lesion detection for focal hepatic lesions at all simulated low-dose levels [8], even at ultra-low-dose levels [37]. By enhancing soft-tissue contrast and preserving structural fidelity, DIR has the potential to support more confident and accurate diagnostic decision-making.

As shown in Fig. (**5**), the colorectal tumor was more conspicuous on DIR than on HIR. DIR emphasizes the visual conspicuity of the tumor by highlighting the hyperattenuating tumor components. Thus, the tumor relationship with surrounding tissue is more distinguishable on DIR images, facilitating clinical diagnosis and therapeutic planning.

### 3.5 Multiregion CT: Achieving Ultra-low-dose for Comprehensive Diagnostic Evaluation

Multiregion CT, such as thoraco-abdomino-pelvic CT for oncologic evaluation and one-stop combined CTA for systemic atherosclerosis assessment, has become increasingly common in clinical practice. However, these protocols are inherently associated with high cumulative radiation exposure due to the large scan range. While modern CT systems have introduced tools such as automatic tube voltage selection and organ-based tube current modulation to reduce radiation dose [38], ultra-low-dose is expected, particularly in patients undergoing repeated multiregion CT scans.

The advanced 'deep' reconstructions offer promising solutions for multiregion CT imaging by enabling high-quality images under ultra-low-dose settings. The application recommendation varies depending on the protocol architecture of multiregion CT, which can be broadly categorized into two types: single-pass multiregion CT and multiregion combined CT scans.

Single-pass multiregion CT refers to protocols where multiple anatomical regions are scanned in a single continuous sequence, *e.g*., thoraco-abdomino-pelvic CT [39]. In preoperative CT of ovarian tumor, where the scan range sometimes even covers from chest to pelvis, DIR is well-suited, especially for reproductive-aged patients. DIR has been reported to enable substantially reduced-dose CT in preoperative imaging of ovarian tumors, while maintaining high diagnostic accuracy for the assessment of peritoneal invasion, lymph node, and hepatic metastasis [27]. Whole-body myeloma CT is another representative example, where DIR can offer significant benefits for radiation dose reduction. As shown in Fig. (**6**), DIR outperformed HIR in visualizing osteolytic lesions at the ultra-low-dose level.

Multiregion combined CT scans consist of multiple acquisition sequences within a single-bolus one-stop examination, each tailored to a specific anatomical region with distinct timing, exposure settings, and reconstruction parameters, *e.g*., one-stop combined CTA of multisite arteries [40-43]. In this scenario, DLR and DIR serve complementary roles. The joint application of DLR on coronary CTA and DIR on aortic CTA has been investigated, which enables 57% reduction of radiation dose in Transcatheter Aortic Valve Implantation (TAVI) planning CT [44]. Similarly, the joint application can be translated to other scenarios that were previously limited by the concern of radiation dose, *e.g*., whole-body CTA for multiterritorial atherosclerosis disease evaluation [45], combined coronary CTA with whole-body CT for clinical management of CAD patients [46].

### 3.6 CT Perfusion: Lowering the Dose Barriers of Functional Imaging

Despite the technical maturity of CT Perfusion (CTP) and proved clinical value in oncology, neurology, and cardiology, the concern of radiation limits its widespread application in clinical practice. There are several well-recognized methods to reduce radiation dose, including lowering tube current, lowering tube voltage, or sparse-view acquisition. However, image quality is degraded with the radiation dose reduction due to increased image noise and streak artifacts.

DLR addresses this issue effectively for myocardial CTP with its cardiac-dedicated nature and efficient reconstruction workflow. The image quality of CTP could be preserved by DLR at low-dose settings, allowing images derived from CTP to maintain diagnostic quality [47]. On the one hand, the coronary CTA from the peak arterial phase of myocardial CTP by using the DLR helps to deliver comparable diagnostic image quality to that of the routine-dose coronary CTA, enabling reliable identification of coronary plaque characteristics and stenosis degree [48]. On the other hand, by lowering the image noise of perfusion maps, DLR has the potential to reduce the variability of perfusion parameters and to improve the consistency and reliability of perfusion analysis [49].

In contrast, DIR is particularly beneficial when perfusion imaging is intended for combined functional and morphological evaluation of the tumor. In the case of renal CTP, where the tumor characterization is essential, DIR is able to provide more conspicuous diagnostic information for tumor staging and adjacent organ invasion at the low dose level. As shown in Fig. (**7**), DIR provides reduced image noise, suppressed artifacts, and improved lesion conspicuity while maintaining consistent perfusion parameters, showing the potential to lower the dose barriers of one-stop-shop evaluation for both function and morphology.

## 4 EMERGING INSIGHTS

### 4.1 Image Texture

Image characteristics, such as artifacts, noise magnitude, and image texture, may vary with different reconstruction algorithms. Trained by thousands of paired high-quality and low-quality datasets, the powerful ability of DLR and DIR on noise magnitude reduction, *i.e*., the standard deviation of CT attenuation within the region of interest, has been well investigated [8, 10, 23, 24]. Moreover, DLR and DIR provide a natural image appearance, which contributes to stable and reliable radiomics. As such, they are deemed to have the potential to improve the reproducibility of the radiomic features that come from repeated scans, repeated measurements, or inter-dose levels. Furthermore, the reproducibility of inter-reconstruction algorithms may help to reveal the robust texture features. As reported in a previous study, the reproducibility of texture features across HIR and DIR demonstrates their robustness [51].

### 4.2 Streak Artifacts

Streak artifacts are commonly encountered in clinical routine. One potential source is photon starvation, which can occur in highly attenuating areas such as the shoulders. Although automatic tube current modulation and adaptive filtration allow sufficient photons to pass through the high-attenuation projection angles or allow more photons to contribute to the reconstruction, the streak artifacts still occur in low-dose settings. Another potential source is incomplete projections, which can be caused by the arm of patients lying outside the scan field of view [12]. Similar effects can also be caused by dense objects, such as an intravenous tube containing contrast medium lying outside the scan field.

The DIR algorithm, which integrates the physics-based model and deep learning-based denoiser, works more effectively in dealing with deteriorated or incomplete projection data to reduce streak artifacts as compared to conventional algorithms [12, 36]. As shown in Fig. (**8**), DIR improves low-contrast lesion detectability by suppressing streak artifacts and reducing image noise, thereby reducing the risk of misdiagnosis in these situations.

## 5 LIMITATIONS AND FUTURE PROSPECTS

Despite the promising advantages mentioned above, several challenges remain for the two 'deep' reconstructions. Methodologically, the inherent “black-box” nature of CNN underlying both DLR and DIR constraints the interpretability, making it difficult for radiologists to understand how specific image features are generated and to develop confidence in their clinical use. Consequently, the assessment of their clinical feasibility should extend beyond image-quality metrics to emphasize diagnostic accuracy, reader confidence, and intra-reader reproducibility. Practically, because the performance of these reconstructions depends heavily on the quality and diversity of the training datasets, characteristic pitfalls such as over-smoothing and the hallucination risk may be introduced when encountering scenarios insufficiently represented in the training dataset. The data-driven nature also limits the generalizability in such insufficiently trained scenarios. Metal artifacts and spectral CT imaging are two representative scenarios where DLR and DIR may underperform or are not yet applicable, representing important directions for future development.

### 5.1 Metal Artifacts

The metal artifacts, which consisted of severe artifacts due to beam hardening and photon starvation [52], can significantly degrade image quality, especially in postoperative evaluation for patients with orthopedic hardware, dental implants, or metallic stents. Due to the data-driven nature of CNN-based reconstructions, the lack of paired images with and without metal artifacts in the training datasets makes these artifacts difficult to be corrected by DLR and DIR.

However, DIR has some potential in reducing metal artifacts, likely owing to its foundation of MBIR, which inherently includes robust modeling of the X-ray acquisition process [53-55]. Nonetheless, neither DLR nor DIR offers a perfect solution for metal artifact reduction at present. This remains an active area of development in the future and may require dedicated algorithms, like an AI-based metal artifact reduction algorithm [56].

### 5.2 Spectral CT

As spectral CT technologies, including Dual-Energy CT (DECT) and Photon-Counting CT (PCCT), continue to gain more and more clinical attention, the integration of 'deep' reconstruction algorithms is expected to play an important role in fully expanding their clinical capabilities.

For DECT, early studies have shown that DLR can enhance the image quality of Virtual Monoenergetic Images (VMIs) by reducing image noise and improving SNR and CNR [57, 58]. Several investigations have also reported that DLR is able to improve the diagnostic performance of VMIs on carotid atherosclerosis and abdominal lesions by improving lesion conspicuity [59, 60]. Moreover, preliminary researches have explored the impact of DLR on the accuracy and variability of iodine quantification, demonstrating its potential benefits in material decomposition [61-63]. These findings support the capability of DLR in improving both qualitative and quantitative evaluation of DECT. In contrast, current DIR implementations are not yet applicable to DECT due to its single-energy iterative framework. However, there is still great potential for the future generations of DIR to realize spectral reconstruction.

As for PCCT, research on 'deep' reconstructions is still at its early stage. Several studies have demonstrated the feasibility of training CNN-based models for noise reduction in post-processing of low-dose PCCT, *e.g*., skeletal CT and coronary CTA [64-67]. Despite these promising results, commercial DLR algorithms have not yet been implemented for clinical PCCT scanners. In the future, the integration of 'deep' reconstructions with PCCT could further enhance image quality, enable further radiation dose reduction, and even help to provide more accurate material decomposition.

## CONCLUSION

The two 'deep' reconstructions reviewed in this article, DLR and DIR, coexist on a 320-row CT platform and are readily available for clinical use. While differences exist between DLR and DIR, both outperformed HIR in their specifically applicable clinical scenarios. These two 'deep' reconstructions are promising tools for delivering CT images with substantially reduced noise, improved spatial resolution, and more natural image texture. In the future, the integration of clinically implemented 'deep' reconstructions with novel CT platform, particularly spectral CT, may represent an important research direction.

## Figures and Tables

**Fig. (1):**
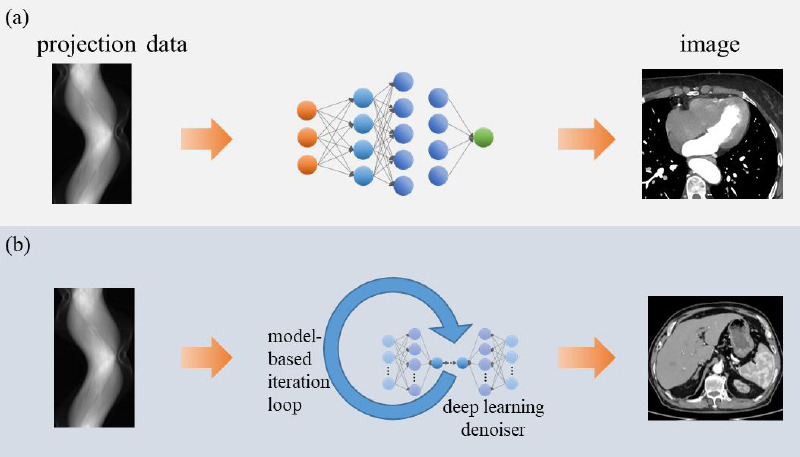
Conceptual illustration of CardioBoost and AIIR. (**a**) CardioBoost, which is specifically designed for cardiac CT imaging, works in a single forward pass, mapping projection data to the image. (**b**) AIIR integrates a deep learning denoiser into the model-based iterative reconstruction loop, iterating between the projection domain and image domain to optimize the final image for non-cardiac body regions.

**Fig. (3):**
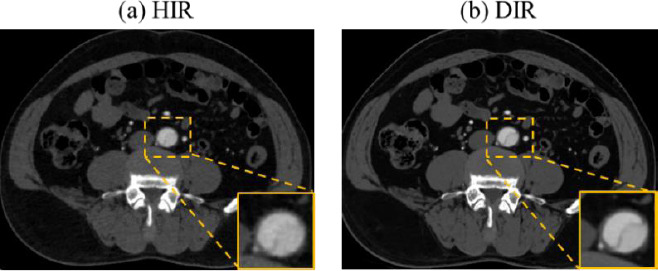
Aortic CTA images obtained in a 49-year-old man with aortic dissection. Images were acquired at a CTDI_vol_ of 8.85mGy and reconstructed with HIR (**a**) and DIR (**b**). The boundary between true lumen and false lumen is more clearly delineated by DIR, at the distal segment involved with the dissection.

**Fig. (4):**
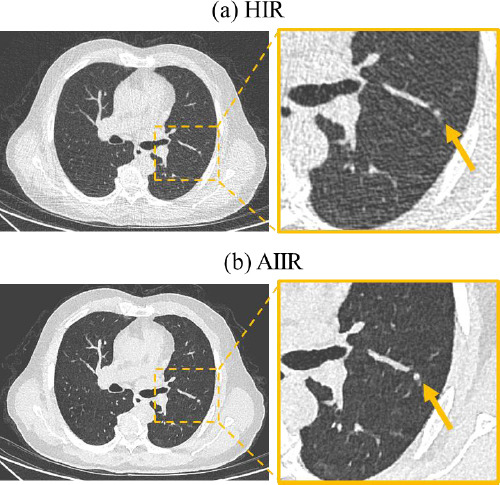
Ultra-low-dose screening chest CT images obtained in a 66-year-old man. Images were acquired at a CTDI_vol_ of 0.22 mGy and reconstructed with HIR (**a**) and DIR (**b**). DIR provides a sharper image appearance than HIR. The nodule (yellow arrow) is clearly visualized on the DIR image, whereas on the HIR image, it is obscured by the noise and streaking artifacts.

**Fig. (5):**
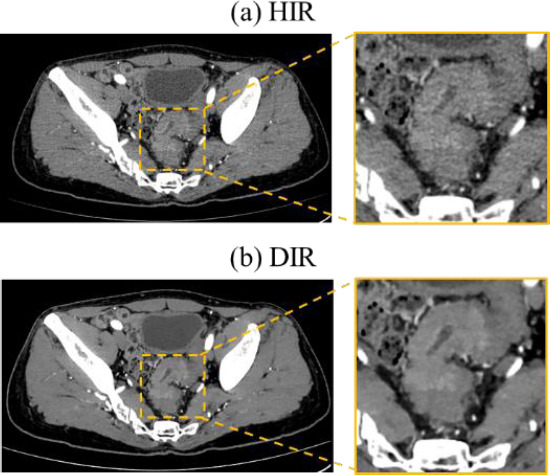
Contrast-enhanced abdominopelvic CT obtained at the arterial phase in a 71-year-old man with colorectal cancer. Images were acquired at a CTDI_vol_ of 12.04 mGy and reconstructed with HIR (**a**) and DIR (**b**). While the routine-dose HIR image is acceptable for the detection of colorectal cancer, the presence of image noise and streak artifacts in the pelvic region hindered the differentiation of the tumor from the surrounding tissue. DIR, in contrast, improves image sharpness and lesion conspicuity, better delineating the tumor boundary and enhancement pattern.

**Fig. (6):**
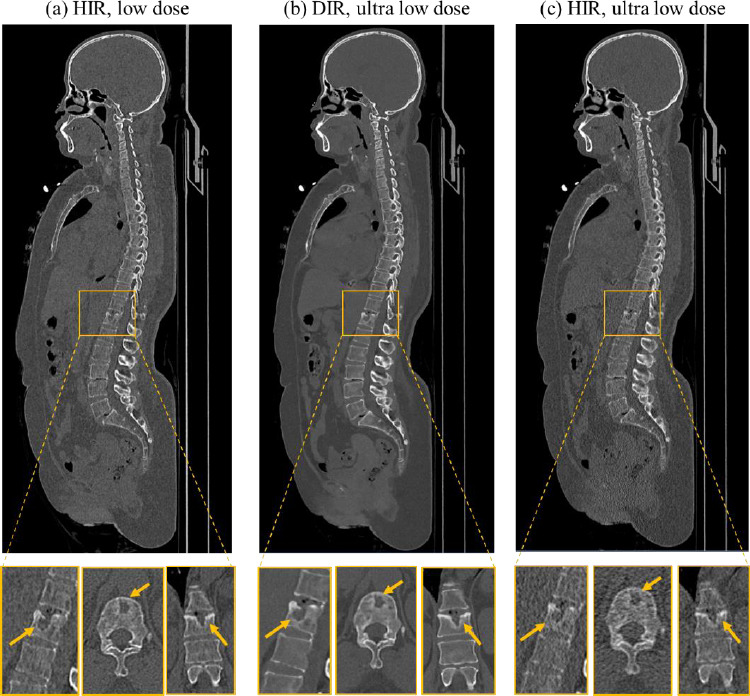
Whole-body CT obtained from a 54-year-old woman with multiple myeloma. Images were acquired at low dose (6.57 mGy) and ultra-low dose (1.39 mGy). The low-dose image was reconstructed with HIR (**a**), and the ultra-low-dose image was reconstructed with DIR (**b**) and HIR (**c**). Detailed graphs of the osteolytic lesion, in the sagittal, axial, and coronal views, are displayed at the bottom. At the ultra-low-dose level, DIR provides optimized visualization of osteolytic lesions as compared with HIR, whereas the vertebral body may appear over-smoothed.

**Fig. (7):**
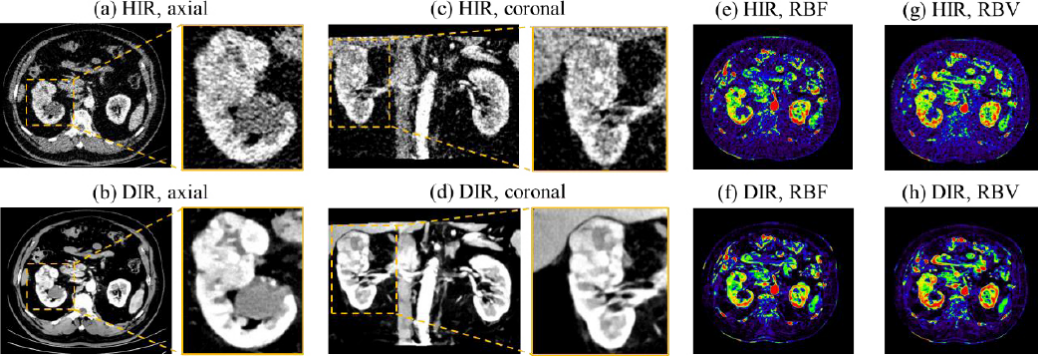
Low-dose volumetric renal CTP obtained from a 69-year-old man with renal tumor. Images were acquired at a CTDI_vol_ of 19.3 mGy and reconstructed with HIR (**a**, **c**, **e**, **g**) and DIR (**b**, **d**, **f**, **h**). The renal tumor originating from the upper pole of the right kidney and the renal cyst in the right kidney are visible on the corticomedullary-phase images (a~d). The lesion details are blurred on HIR images due to substantial image noise (**a**, **c**). With improvement in spatial resolution and noise reduction, DIR images yielded better delineation of the tumor margin and relationship with the adjacent liver (**b**, **d**). The necrotic core of the renal tumor is also better delineated on DIR images (**b**, **d**). While improving anatomical characterization for low-dose renal CTP, DIR maintained the perfusion maps of Renal Blood Flow (RBF) and Renal Blood Volume (RBV) consistent with HIR (e~h).

**Fig. (8):**
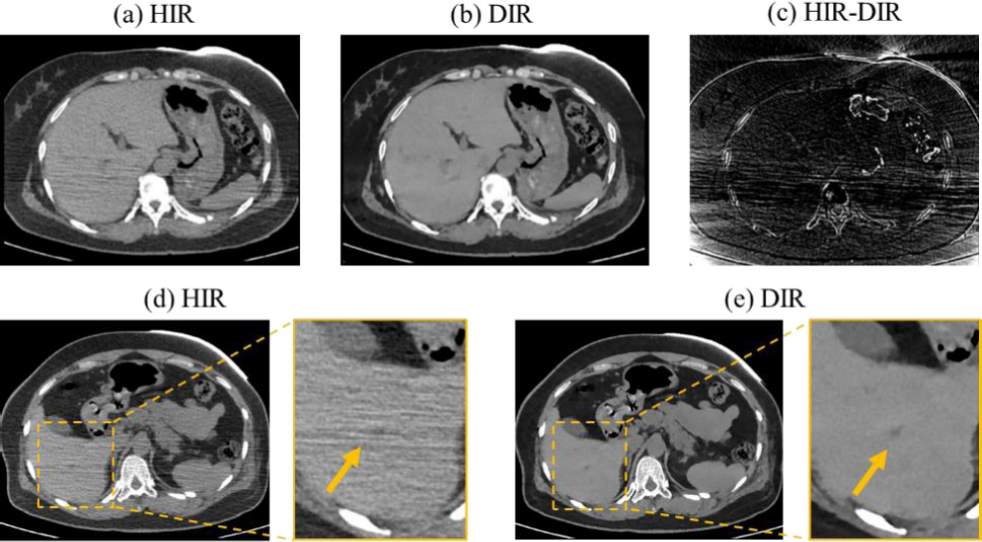
Non-contrast abdominal CT obtained in a 53-year-old woman with irregular arm positioning. Images were acquired at 9.322 mGy and reconstructed with HIR and DIR. At the level with the most severe artifacts (**a**~**c**), image noise and streak artifacts are reduced by DIR. Subtraction images of HIR-DIR (**c**) reveal no loss of anatomical details aside from bone structures, indicating that anatomical detail has been well preserved. At another axial level (**d**, **e**), the hypoattenuating liver cyst is obscured by the severe streak artifacts on the HIR images but sharply delineated on the DIR image.

**Table 1:** Detailed parameters of the two reconstruction algorithms.

**Parameters**	**CardioBoost**	**AIIR**
Network structure	CNN	MBIR incorporated with CNN
Algorithm type	Operate on the projection domain	Operate on the projection domain
Iteration	N/A	Between the projection domain and the image domain
Regularization	N/A	Deep-learning-based regularization
Training dataset	Paired high-quality and low-quality cardiac CT datasets, including anthropomorphic phantom and clinical cases	Paired high-quality and low-quality datasets across various body parts, including anthropomorphic phantom and clinical cases
Ground truth images	FBP	MBIR
Kernel type	Cardiac	Head; body; lung; bone;…
Resolution type	Soft; standard; sharp	Soft; standard; sharp
Anatomical region	Heart	Whole body except from heart
Clinical scenarios	CSCT; CCTA; MCTP	Contrast-enhanced and non-enhanced CT; CTA; CTP
Scan modes	Volume and helical	Volume and helical

**Abbreviation:** CSCT: coronary calcium scoring CT; MCTP: myocardial CT perfusion.

**Table 2:** Quantitative comparison between HIR and deep learning reconstructed images.

**Fig No.**	**Area Measured (Target; Background)**	**Image Noise**		**SNR**		**CNR**	
		**HIR**	**DLR/DIR**	**HIR**	**DLR/DIR**	**HIR**	**DLR/DIR**
Fig.2, a and d	LCX; epicardial fat	26.0	9.6	14.7	21.5	15.12	48.4
Fig.3, a and b	aorta; muscle	13.6	7.7	28.6	48.9	19.3	35.5
Fig.4	pulmonary parenchyma; intratracheal gas	90.4	43.8	9.9	13.6	0.9	2.0
Fig.5	rectal tumor; muscle	12.6	6.6	3.7	8.6	0.2	1.0
Fig.6	osteolytic lesion; normal bone	165.3	58.7	0.3	1.1	0.8	2.1
Fig.7	healthy renal cortex; muscle	44.1	16	3.4	9.1	3.3	9.1

**Note:** Image noise refers to the Standard Deviation (SD) of the background tissues. SNR= CT_target _/SD_target_. CNR=|CT_target_-CT_background_|/SD_background_.

**Table 3:** Some studies about the clinical application of DLR and DIR on CT imaging.

**Refs.**	**Scenario**	**Algorithm**	**Study Type**	**Image Groups**	**Image Quality Performance**	**Diagnostic Performance**
[9]	CSCT	DLR	Patient	RD-HIR, LD-DLR, ULD-DLR	Compared to RD-HIR, LD-DLR and ULD-HIR produced comparable or lower image noise and higher CNR	DLR allowed accurate calcium scoring and reliable cardiovascular risk categorization at LDCT and ULDCT.
[23]	CTA	DIR	Patient	RD-FBP, RD-HIR, RD-DIR	Compared to HIR, DIR reduced image noise by 35%~45%, and improved SNR and CNR by 70%~90%.	DIR provided superior visualization of vascular structures and pulmonary emboli compared with both FBP and HIR.
[36]	Chest CT	DIR	Patient	RD-HIR, LD-HIR, LD-DIR	Compared to HIR, DIR reduced the image noise by ~64%.	LD-DIR yielded pneumonia severity index and airway stenosis grading more consistent with RD-HIR than LD-HIR.
[10]	Abdominal CT	DIR	Patient	RD-HIR, RD-DIR	Compared to HIR, DIR offered significantly higher SNRs and CNRs, and more conspicuous imaging features	Compared to HIR, DIR improves the diagnostic performance regarding colorectal cancer
[26]	Abdominal CT	DIR	Phantom and patient	RD-HIR, LD-HIR, LD-DIR	Compared to HIR, DIR exhibited higher TTF_50%_, reduced NPS noise magnitude, and comparable f_av_ in the phantom studyCompared to RD-HIR, LD-DIR has lower image noise, higher SNR, and CNR in clinical cases	None
[44]	TAVI planning CT	DLR+DIR	Patient	RD-HIR, LD-DIR, LD-DLR	Compared to RD-HIR, LD-DLR showed 21%–55% lower image noise and 63%-79% higher CNR of coronary arteriesCompared to RD-HIR, LD-DLR showed 48%–53% lower image noise and 79%-114% higher CNR of aortoiliac arteries	Reduced-dose CT with joint application of DLR and DIR is feasible for assessing aortic valve morphology and determining vascular access routes for TAVI planning.
[50]	Cerebral CTP	DIR	Patient	RD-HIR, LD-DIR	Compared to RD-HIR, CTP-derived LD-DIR achieved significantly higher SNR and CNR	Compared to RD-HIR, CTP-derived LD-DIR achieved comparable diagnostic accuracy in identifying the responsible vessels of AIS

**Abbreviations:** CSCT: Coronary Calcium Scoring CT; RD-HIR: HIR images acquired at routine dose; RD-FBP: FBP images acquired at routine dose; LD-HIR: HIR images acquired at low dose; LD-DLR: DLR images acquired at low dose; LD-DIR: DIR images acquired at low dose; ULD-DLR: DLR images acquired at ultra-low dose; LDCT: Low Dose CT; ULDCT: Ultra-Low Dose CT; TTF_50%_: frequency at the task transfer functions reached 50%; NPS: noise power spectra; f_av_: average spatial frequency of the NPS curve TAVI: Transcatheter Aortic Valve Implantation; AIS: Acute Ischemic Stroke.

## LIST OF ABBREVIATIONS

DLR: Deep Learning Reconstruction
DIR: Deep Iterative Reconstruction
HIR: Hybrid Iterative Reconstruction
FBP: Filtered Back Projection
MBIR: Model-Based Iterative Reconstruction
CNN: Convolutional Neural Network
AIIR: Artificial Intelligence Iterative Reconstruction
CTA: CT Angiography
CTP: CT Perfusion
DECT: Dual-Energy CT
PCCT: Photon-Counting CT
SNR: Signal-to-Noise Ratio
CNR: Contrast-to-Noise Ratio
